# Pre-diagnostic and non-advanced systemic mastocytosis without cutaneous involvement have an increased risk of anaphylaxis

**DOI:** 10.3389/falgy.2025.1681051

**Published:** 2025-10-17

**Authors:** Andrea Sangalli, Valerio Pravettoni, Mariarita Sciumè, Dario Consonni, Silvio Sartorio, Nicola Montano, Federica Rivolta

**Affiliations:** 1 General Medicine Unit, Department of Internal Medicine, Fondazione IRCCS Ca’ Granda, Ospedale Maggiore Policlinico, Milan, Italy; 2 Hematology Unit, Department of Internal Medicine, Fondazione IRCCS Ca’ Granda Ospedale Maggiore Policlinico, Milan, Italy; 3 Occupational Health Unit, Fondazione IRCCS Ca’ Granda Ospedale Maggiore Policlinico, Milan, Italy; 4 Allergy and Clinical Immunology Residency, University of Milan, Milan, Italy; 5 Department of Clinical Sciences and Community Health, University of Milan, Milan, Italy

**Keywords:** anaphylaxis, anaphylaxis risk factors, hymenoptera venom allergy, mast cell disease, pre-diagnostic systemic mastocytosis, systemic mastocytosis

## Abstract

**Background:**

Patients with mastocytosis have a higher risk of anaphylactic reactions. This study aims to assess the prevalence and risk factors of anaphylaxis among patients diagnosed with Systemic Mastocytosis (SM), including pre-diagnostic Systemic Mastocytosis (pre-SM), a subgroup of patients often overlooked in current classifications.

**Methods:**

A retrospective monocentric study was conducted at Fondazione IRCCS Ca’ Granda Ospedale Maggiore Policlinico in Milan, Italy. Patients aged ≥18 years diagnosed with SM or pre-SM between January 2009 and May 2025 were included. Demographic, clinical and laboratory data were analyzed using chi-squared test or Wilcoxon-Mann–Whitney and Kruskal–Wallis tests

**Results:**

At the time of diagnosis, out of 162 patients (53% women), 29 (18%) experienced at least one episode of anaphylaxis. Hymenoptera venom was the main trigger (51.7%), followed by drugs (27.6%) and idiopathic cases (20.7%). Patients with anaphylaxis had 7% pre-SM, 48% BMM, 28% ISM, 0% SSM, 7% ASM, 10% SM-AHN, (*p* < 0.001). The prevalence of anaphylaxis in each subtype was as follows: 2/12 (17%) in pre-SM, 14/31 (45%) in BMM, 8/97 (8%) in ISM, 0/5 in SSM, 2/4 (50%) in ASM and 3/13 (23%) in SM-AHN, (*p* < 0.001). Hymenoptera venom–induced anaphylaxis occurred exclusively in indolent forms (pre-SM, BMM, and ISM) while drug-induced anaphylaxis was observed in both ISM and advanced SM subtypes. Idiopathic anaphylaxis was more evenly distributed across all SM subtypes, (*p* < 0.001). The presence of cutaneous lesions was associated with a lower risk of anaphylaxis: 10/114 (8.8%) vs. 19/48 (39.6%) without skin involvement (*p* < 0.001), with a confirmed protective effect in both ISM and pre-SM. Male sex was identified as an additional risk factor, (*p* = 0.03). A history of Hymenoptera sting was associated with a higher risk of Hymenoptera venom anaphylaxis: 15/113 (13%) vs. no reactions to the first sting in 47 patients, (*p* = 0.011).

**Conclusion:**

Anaphylaxis is a relevant issue not only in acknowledged variants of SM, but also in pre-diagnostic forms. Idiopathic anaphylaxis may occur across different subtypes. Hymenoptera venom is the main trigger in indolent forms, whereas drug-induced reactions predominate in ISM and advanced SM, mainly through IgE-independent mechanisms. The risk of anaphylaxis is higher in pre-SM and ISM without cutaneous involvement, particularly in case of Hymenoptera venom sensitization. Our results highlight the need for allergological risk assessment and close monitoring especially in patients without skin lesions or with Hymenoptera venom sensitization.

## Introduction

Mastocytosis comprises a heterogeneous group of rare clonal neoplasms characterized by the proliferation and accumulation of abnormal mast cells in one or more organ systems (1).

The latest World Health Organization (WHO) classification confirmed the established distinction between cutaneous mastocytosis (CM), systemic mastocytosis (SM), and the rare entity mast cell sarcoma (2).

CM is confined to the skin and predominantly affects pediatric patients, with a favorable prognosis and a significantly lower risk of anaphylaxis compared to SM (3).

SM is defined by the involvement of at least one internal organ, which may be accompanied by cutaneous lesions. The classification into subtypes is useful from a haematological perspective, especially regarding prognosis: non-advanced variants such as Bone Marrow Mastocytosis (BMM), Indolent SM (ISM) and Smoldering SM (SSM) have a higher survival rate compared to advanced forms such as Aggressive SM (ASM), SM with an Associated Hematopoietic Neoplasm (SM-AHN) and Mast Cell Leukemia (MCL) (4).

Patients who do not meet the diagnostic criteria for SM but fulfil one or two minor criteria of monoclonal origin are classified as having pre-diagnostic Systemic Mastocytosis (pre-SM) or monoclonal mast cell activation syndrome (MMCAS) depending on the presence or absence of mast cell activation symptoms (5, 6).

The clinical presentation of mastocytosis is heterogeneous, ranging from symptoms due to cutaneous involvement to signs related to inappropriate mast cell mediator release (e.g., allergic reactions including anaphylaxis, osteopenia and osteoporosis). One hypothesis attributes the link between SM and anaphylaxis to the hyperactive state of mast cells carrying the activating D816V mutation, another one considers the increased number of potential effector cells as a factor increasing the risk of immediate-type allergic reactions (3). The risk of anaphylaxis is higher in ISM and BMM subtypes (7). Advanced forms of SM (advSM), characterized by extensive tissue infiltration, may manifest with organ dysfunction and often require cytoreductive therapy (1).

Considering the complexity of the disease, a multidisciplinary approach is essential, involving hematologists, allergists, dermatologists, endocrinologists, and gastroenterologists. Treatment options vary according to clinical presentation, ranging from symptomatic therapy (anti-mediators) and anaphylaxis prevention (e.g., emergency kit with epinephrine, patient education, venom immunotherapy when appropriate), to treatment of osteopenia or osteoporosis, and cytoreductive treatment (8).

Allergy evaluation is recommended as patients with SM have an approximately 100-fold increased risk of anaphylaxis compared to the general population. Given the rarity of the disease, the prevalence of anaphylactic reactions is estimated to range widely from 20% to 56%. The main cause of anaphylaxis is Hymenoptera venom (HV)—particularly wasp stings—followed by idiopathic anaphylaxis (with no identified cause) and lastly, drugs and foods (9).

Certain clonal mast cell disease (MCD) such as BMM, ISM and MMCAS are established risk factors for severe Hymenoptera sting–induced anaphylaxis. Hereditary alpha tryptasemia (HαT), a genetic trait characterized by increased copy numbers of TPSAB1 gene encoding alpha-tryptase and consequently by basal serum tryptase level above 8 μg/L, is also associated with severe Hymenoptera venom allergy (HVA), with the risk further increased in the presence of concomitant clonal MCD (10).

According to the 2022 WHO classification update, which recognized BMM as a distinct subtype (2), this monocentric study aimed to evaluate the prevalence of anaphylaxis in a group of patients with SM or pre-SM. Anaphylaxis triggers and possible risk or protective factors for anaphylaxis were analyzed across different SM subtypes, including emerging forms such as pre-SM, to stratify allergological risk.

## Methods

A retrospective monocentric study group was conducted at the Allergology Clinic of the Foundation IRCCS Ca' Granda Ospedale Maggiore Policlinico in Milan, Italy, where patients with SM are managed by a multidisciplinary team including allergists, hematologists, dermatologists and endocrinologists.

Patients aged 18 years or older were included if diagnosed with SM according to latest WHO criteria (2) or pre-SM, defined as a clonal abnormal bone marrow mast cell infiltrate that does not meet full diagnostic criteria for SM. Markers of clonality are defined by the presence of KIT D816V mutation in bone marrow or in peripheral blood (digital or ASO-PCR, Sanger sequencing and next generation sequencing) and/or the aberrant expression of CD25/CD2 on mast cells on multiparameter flow cytometry.

Data were collected in a strictly pseudonymized manner using a case report form on the Research Electronic Data Capture (REDCap®) platform, which is validated in accordance with national regulations.

Clinical data were extracted from the electronic medical records of SM patients followed between January 2009 and May 2025, and included:

– Socio-demographic variables: sex, age at diagnosis,
– Anamnestic and laboratory data: SM subtypes by diagnostic criteria [presence of major and minor WHO criteria (2)], comorbid conditions (rhinitis, bronchial asthma, atopic dermatitis, food allergy), presence of cutaneous lesions, history of Hymenoptera stings, history of anaphylaxis at diagnosis and associated triggers identified through an allergological workup. HV allergy was confirmed by positive skin tests and/or serum IgE for venom extracts and for recombinant allergens (8) (ImmunoCAP system, Thermo Fisher Scientific®), food allergy by skin prick test (Lofarma® and Stallergenes®) and serum IgE for food and for recombinant allergens (ImmunoCAP system, Thermo Fisher Scientific®), drug allergy by serum specific IgE for beta-lactams (ImmunoCAP®, Thermo-Fisher), cutaneous tests (DAP, DIATER Laboratories®), basophil activation test (BAT) and drug provocation test. Idiopathic anaphylaxis was diagnosed if all potential triggers had been ruled out (3).

We calculated anaphylaxis prevalence and 95% confidence interval (CI). Associations between a history of anaphylaxis and clinical or laboratory variables were analyzed using the chi-squared tests.

Age at diagnosis and triptase levels were analyzed using Wilcoxon-Mann–Whitney and Kruskal–Wallis tests. Statistical analysis was performed using Stata 18 software (StataCorp, 2023).

The study received approval from the Ethics Committee of the Fondazione IRCCS Ca' Granda Ospedale Maggiore Policlinico (5916_16.04.2025_P). It was conducted in accordance with the principles of Good Clinical Practice, the ethical guidelines of the Declaration of Helsinki, and current regulations on observational studies.

## Results

### Study group

The study group included 162 adult patients, comprising 86 women (53.1%) and 76 men (46.9%). The median/mean age at diagnosis was 49.5/50.8 years (range 22–86 years, SD 15.3).

Patients were classified into the following diagnostic subtypes: 12 (7.4%) pre-SM, 31 (19.1%) BMM, 97 (59.9%) ISM, 5 (3.1%) SSM, 4 (2.5%) ASM, and 13 (8.0%) SM-AHN.

Median and mean age were lower in patients with ASM and pre-SM, and was higher in patients with SM-AHN and SSM. Pre-SM, BMM and SSM were more frequently diagnosed in male patients, whereas ISM was more common in females.

The overall prevalence of atopy was 27.8%, with 37 of 162 patients (22.8%) affected by allergic rhinitis, 7 (4.3%) by allergic asthma, and 1 (0.6%) by atopic dermatitis. No significant differences were observed in the prevalence of allergic diseases among the different subtypes.

Cutaneous involvement due to mastocytosis was more frequently observed in ISM patients, but a prevalence above 60% was also seen in pre-SM, SSM, and ASM.

Mastocytosis diagnostic criteria showed a heterogeneous distribution across the different subtypes, except for the KIT D816V mutation, which was homogeneously expressed (Table 1).

**Table 1:** Characteristics of the study group by subtype.

Characteristics	Pre-SM	BMM	ISM	SSM	ASM	SM-AHN	
	*N* = 12	*N* = 31	*N* = 97	*N* = 5	*N* = 4	*N* = 13	*p* value
Median/mean age at diagnosis (range), years	40/45.3 (22–84)	58/54.3 (31–73)	45/47.4 (22–86)	74/74.4 (67/82)	34/39.8 (29–62)	69/66.8 (36–83)	<0.001
Male/female	8/4	19/12	35/62	4/1	2/2	8/5	0.03
Allergic diseases, no. (%)							
Rhinitis	6 (50%)	5 (16%)	22 (23%)	1 (20%)	0	3 (23%)	NS
Asthma	0	1 (3%)	4 (4%)	0	0	2 (15%)	NS
Atopic Dermatitis	0	0	1 (1%)	0	0	0	NS
Food Allergy	0	0	0	0	0	0	NA
Cutaneous involvement, no. (%)	8 (67%)	0	95 (98%)	3 (60%)	3 (75%)	5 (38%)	<0.001
Median/mean basal serum tryptase level (range), μg/L	12.5/14.6 (4–34)	23/29.1 (8–102)	24/35.5 (5–227)	182/212.2 (22–484)	195.5/259 (45–600)	37/64.2 (18–200)	<0.001
SM diagnostic criteria							
Presence of major criterion, no. (%)	0	17 (55%)	47 (48%)	4 (80%)	4 (100%)	9 (69%)	0.001
Abnormal bone marrow MC morphology (>25% of bone marrow MCs), no. (%)	1 (8%)	26 (84%)	85 (88%)	5 (100%)	4 (100%)	10 (77%)	<0.001
CD25/CD2/CD30 abnormal expression, no. (%)	0	30 (97%)	93 (96%)	5 (100%)	4 (100%)	12 (92%)	<0.001
Positive KITD816V mutation on bone marrow or peripheral blood, no. (%)	12 (100%)	26 (84%)	74 (76%)	4 (80%)	3 (75%)	10 (77%)	NS
Tryptase level ≥ 20 ng/ml, no. (%)	2 (17%)	20 (65%)	63 (65%)	5 (100%)	4 (100%)	12 (92%)	0.001

Pre-SM, pre-diagnostic systemic mastocytosis; BMM, bone marrow mastocytosis; ISM, indolent systemic mastocytosis; SSM, smoldering systemic mastocytosis; ASM, aggressive systemic mastocytosis; SM-AHN, SM with an associated hematopoietic neoplasm; SM, systemic mastocytosis; N, numerosity; NS, not significant; NA, not applicable.

### Anaphylaxis prevalence and triggers

At the initial evaluation, at least one episode of anaphylaxis was reported in 29 patients (17.9%), of whom 19 (65.5%) were male and 10 (34.5%) were female (*p* = 0.03).

The median/mean age at diagnosis was 58/52.7 years (range 29–78, SD 13.2) in the group with anaphylaxis and 49/50.3 (range 22–86, SD 15.7) in the group without anaphylaxis (*p* = 0.38).

The identified triggers of anaphylaxis included Hymenoptera venom in 15 of 29 cases (51.7%), drugs in 8 of 29 (27.6%), and idiopathic causes in 6 of 29 (20.7%).

Hymenoptera venom–induced anaphylaxis occurred exclusively in indolent forms of SM (pre-SM, BMM, and ISM), drug-induced anaphylaxis was observed in both ISM and advanced SM subtypes, while idiopathic anaphylaxis showed a more homogeneous distribution across SM subtypes (Table 2).

**Table 2:** Anaphylaxis triggers in systemic mastocytosis subtypes.

Subtype	Anaphylaxis triggers			Total
	Hymenoptera venoms	Drugs	None (idiopathic)	
Pre-SM	1 (50.0%)	0	1 (50.0%)	2 (100%)
BMM	12 (85.7%)	0	2 (14.3%)	14 (100%)
ISM	2 (25.0%)	5 (62.5%)	1 (12.5%)	8 (100%)
SSM	0	0	0	0
ASM	0	1 (50.0%)	1 (50.0%)	2 (100%)
SM-AHN	0	2 (66.7%)	1 (33.3%)	3 (100%)
*p* < 0.001				

Pre-SM, pre-diagnostic systemic mastocytosis; BMM, bone marrow mastocytosis; ISM, indolent systemic mastocytosis; SSM, smoldering systemic mastocytosis; ASM, aggressive systemic mastocytosis; SM-AHN, SM with an associated hematopoietic neoplasm.

Wasp venom was the most frequent cause of HVA, accounting for 13 of 15 cases (87.7%). One patient (6.7%) experienced anaphylaxis due to bee venom, and another (6.7%) reacted to both wasp and bee venom. In one case (6.7%), the culprit insect could not be identified and standard diagnostic tests yielded negative results.

Involved drugs in 8 patients were acetylsalicylic acid (2), ibuprofen (1), ketorolac (1), amoxicillin (1), cephazolin as pre-anesthesia prophylaxis (1), intravenous iron preparation (1), intramuscular cyanocobalamin (1).

No association was found between the presence of specific allergic comorbidities and the occurrence of anaphylaxis.

### Anaphylaxis and SM subtypes

Out of 29 cases of anaphylaxis 2 (6.9%) were registered in pre-SM, 14 (48.3%) in BMM, 8 (27.6%) in ISM, 0 (0%) in SSM, 2 (6.9%) in ASM, and 3 (10.3%) in SM-AHN (*p* < 0.001).

The prevalence of anaphylaxis was associated with subtype (*p* < 0.001, Table 3), with anaphylaxis around 50% in patients with BMM and ASM.

**Table 3:** Anaphylaxis prevalence in systemic mastocytosis subtypes.

Subtype	Anaphylaxis		Total
	No	Yes	
Pre-SM	10 (83.3%)	2 (16.7%)	12 (100%)
BMM	17 (54.8%)	14 (45.2%)	31 (100%)
ISM	89 (91.7%)	8 (8.3%)	97 (100%)
SSM	5 (100%)	0	5 (100%)
ASM	2 (50.0%)	2 (50.0%)	4 (100%)
SM-AHN	10 (76.9%)	3 (23.1%)	13 (100%)
*p* < 0.001			

Pre-SM, pre-diagnostic systemic mastocytosis; BMM, bone marrow mastocytosis; ISM, indolent systemic mastocytosis; SSM, smoldering systemic mastocytosis; ASM, aggressive systemic mastocytosis; SM-AHN, SM with an associated hematopoietic neoplasm.

The median/mean basal serum tryptase level was 25/43.5 μg/L (range: 4–484; SD: 61.0) in patients without anaphylaxis, compared to 26/57.4 μg/L (range: 6–600; SD: 111.2) in those with anaphylaxis (*p* = 0.97). No significant differences were found between the group with or without anaphylaxis with respect to comorbid atopic diseases or SM diagnostic criteria.

Anaphylaxis occurred in 10 of 114 patients (8.8%) with cutaneous lesions compared to 19 of 48 (39.6%) without skin involvement (*p* < 0.001).

Among ISM patients, 6 of 95 (6.3%) with cutaneous lesions experienced anaphylaxis vs. 2 of 2 (100%) without lesions (*p* < 0.001). In the pre-SM group, none of the 8 patients with skin lesions developed anaphylaxis, compared to 2 of 4 (50%) without lesions (*p* = 0.03). No significant association between cutaneous involvement and anaphylaxis was observed in patients with advSM (Table 4).

**Table 4:** Anaphylaxis prevalence in systemic mastocytosis subtypes according to cutaneous involvement.

Subtype	Anaphylaxis		Total	*p* value
	No	Yes		
Pre-SM				0.03
No CI	2 (50.0%)	2 (50.0%)	4 (100%)	
Yes CI	8 (100%)	0	8 (100%)	
BMM				NA
No CI	17 (54.8%)	14 (45.2%)	31 (100%)	
ISM				<0.001
No CI	0	2 (100%)	2 (100%)	
Yes CI	89 (93.7%)	6 (6.3%)	95 (100%)	
SSM				
No CI	2 (100%)	0	2 (100%)	
Yes CI	3 (100%)	0	3 (100%)	
ASM				0.25
No CI	1 (100%)	0	1 (100%)	
Yes CI	1 (33.3%)	2 (66.7%)	3 (100%)	
SM-AHN				0.25
No CI	7 (87.5%)	1 (12.5%)	8 (100%)	
Yes CI	3 (60%)	2 (40%)	5 (100%)	

CI, cutaneous involvement; Pre-SM, pre-diagnostic systemic mastocytosis; BMM, bone marrow mastocytosis; ISM, indolent systemic mastocytosis; SSM, smoldering systemic mastocytosis; ASM, aggressive systemic mastocytosis; SM-AHN, SM with an associated hematopoietic neoplasm; NA, not applicable.

### Hymenoptera venom anaphylaxis and SM subtypes

HV anaphylaxis was more frequent in male patients (11 of 76; 14.5%) compared to females (4 of 86; 4.7%) (*p* = 0.031).

The median/mean age at diagnosis was 59/54.2 years (range: 33–67; SD: 11.0) in the anaphylaxis group and 49/50.4 years (range: 22–86; SD: 15.7) in the non-anaphylaxis group (*p* = 0.30).

Among those with HV anaphylaxis, 12 of 15 patients (80%) had BMM, 2 (13.3%) had ISM, and 1 (6.7%) had pre-SM.

Of the 113 patients with a documented history of a Hymenoptera sting, 15 (13.3%) developed anaphylaxis after a subsequent sting, compared to none of the 49 patients who had never been stung (*p* = 0.007).

Of the 162 patients, 132 underwent testing for HV sensitization. Among these, 13 of 43 sensitized individuals (30.2%) experienced HV anaphylaxis, vs. 2 of 89 (2.3%) who tested negative to standard diagnostic tests (*p* < 0.001).

The median/mean baseline serum tryptase level was 17/23.8 μg/L (range: 8–102; SD: 23.4) in the HV anaphylaxis group, compared to 26/48.3 μg/L (range: 4–600; SD: 75.3) in the non-HV anaphylaxis group (*p* = 0.03).

No differences in the prevalence of concomitant atopic diseases or SM diagnostic criteria were observed between groups.

HV anaphylaxis occurred in 2 of 114 patients (1.8%) with cutaneous lesions vs. 13 of 48 (27.1%) without skin involvement (*p* < 0.001).

## Discussion

At the time of SM diagnosis, the overall prevalence of at least one episode of anaphylaxis was 17.9%, comparable to the 22% reported by a Spanish case series of the Red Espanola De Mastocitosis (REMA) group, which evaluated 163 adults with mastocytosis (11). Other studies have documented a higher prevalence of anaphylaxis in SM patients, ranging from 43% to 73% (12, 13). These discrepancies may stem from differences in patient selection—particularly in studies where many SMs were diagnosed following anaphylaxis—or from broader definitions of systemic reactions, including those limited to mucosal or cutaneous manifestations.

HV was identified as the main trigger of anaphylaxis (52%). Wasp venom was the most frequent cause. This finding confirms previous reports identifying Hymenoptera venom as the leading trigger of anaphylaxis in SM (11). The strong association between a history of Hymenoptera sting and HV anaphylaxis—confirmed by positive HV sensitization tests in most cases—supports an IgE-mediated mechanism.

Drugs represented the second most common trigger (28%), while 21% of anaphylaxis cases were idiopathic. No food-induced anaphylaxis was recorded. These results align with prior literature (3). One study reporting a higher frequency (24%) of food-related anaphylaxis attributed most reactions to alcohol ingestion, without evidence of food allergen sensitization, suggesting a nonspecific, mast cell–mediated activation mechanism (14).

Male sex remained a significant risk factor for anaphylaxis of all causes, consistent with the findings of the REMA group (11). We also observed a male predominance in HV anaphylaxis, although less pronounced than the strong correlation reported by Alvarez-Twose and colleagues in patients with ISM and HV reactions (15). As the author himself points out, it remains unclear whether male sex is an intrinsic predisposing factor or reflects increased occupational or recreational exposure to Hymenoptera stings (16).

The absence of a marked male predominance in all-cause anaphylaxis and the relatively low mean basal serum tryptase levels (<25 μg/L) in HV anaphylaxis support recent evidence indicating reduced sensitivity of the REMA score in detecting clonal MCD (10).

Atopic diseases had a prevalence of 28%, consistent with rates in the general population, and did not constitute a risk factor for anaphylaxis (11, 14).

A protective factor against anaphylaxis—both in ISM and pre-SM—was the presence of cutaneous involvement by the disease, even in patients with HV anaphylaxis. This observation corroborates findings from multiple case series (9, 14, 15).

In our study, anaphylaxis was frequently observed in recognized forms of SM, except for SSM, and also occurred in pre-SM. Notably, 17% of patients with pre-SM experienced anaphylaxis, triggered by HV or of idiopathic origin. As early as 2007, a subset of patients initially diagnosed with idiopathic anaphylaxis were found to harbor aberrant mast cells with clonal markers (17). Consequently, some authors proposed applying similar clinical management for pre-diagnostic forms as for SM, including anaphylaxis prevention, anti-mediator therapy, assessment for bone disease, and close monitoring for potential disease progression (5, 18). Our findings suggest that pre-SM warrants recognition in the current classification of SM, which remains absent in both the latest 2022 WHO and International Consensus Classification (ICC) updates (2, 19).

Although the elevated risk of anaphylaxis in ISM and BMM is well established (7), our data indicate that this risk is not negligible in advanced subtypes either, though it may present with distinct characteristics.

HV-induced anaphylaxis was exclusively observed in indolent forms—most frequently in BMM, followed by ISM and pre-SM. The CEREMAST study group reported an even higher rate of HV-induced anaphylaxis in patients with bone marrow clonal mast cells who did not meet SM or mast cell activation syndrome (MCAS) diagnostic criteria, emphasizing the need for tailored monitoring and management (20). One hypothesis to explain the higher prevalence of HVA in non-advSM is the increased susceptibility to IgE-mediated reactions in the context of a relatively low mast cell burden.

In contrast, drug-induced anaphylaxis occurred in both ISM and advSM, particularly in response to non-steroidal anti-inflammatory drugs (NSAIDs; 4/8, 50%) and beta-lactams (BLs; 2/8, 25%). The remaining cases involved intravenous iron and subcutaneous cyanocobalamin. Most of these reactions were likely due to IgE-independent mechanisms, leading to direct mast cell degranulation (21, 22). Interestingly, the ECNM registry identified a higher incidence of drug-induced hypersensitivity in advSM. The most frequent triggers were NSAIDs and BLs, with elevated serum tryptase levels identified as a significant risk factor for drug reactions. As previously hypothesized by the authors of the ECNM registry (23), the greater likelihood of drug-induced anaphylaxis in advanced forms may result from increased medication exposure in patients with aggressive hematologic diseases, and from the potential direct mast cell activation, especially in a context where cytoreductive therapies are frequently used.

Idiopathic anaphylaxis occurs across all SM subtypes without a specific distribution pattern (3).

It is clear that mastocytosis is not a homogeneous disorder, therefore, its subtypes require precise characterization and individualized risk stratification (24).

A particularly underrecognized subtype is pre-SM, which is associated with an increased risk of severe allergic reactions, bone involvement, and mast cell–mediated symptoms (25, 26). As such, pre-SM should be evaluated and monitored similarly to other non-advanced forms of SM, in order to prevent complications, such as anaphylaxis and osteoporosis (24, 27).

A strenght of the study is the recruitment of patients by a multidisciplinary team made up of haematologist, allergist, dermatologist, endocrinologist and gastroenterologist which allowed us to have a heterogeneous population of patients affected by systemic mastocytosis. The retrospective design of the study allowed the inclusion of a relatively large number of patients with SM and pre-SM, which is essential to study a rare disease. The analysis succeeded in identifying subgroups of patients with specific clinical characteristics associated with a higher risk of anaphylaxis, aiding in patient risk stratification and guiding personalized care.

Nonetheless, the study has several limitations inherent to its observational nature. First, the reliance on medical records may have led to incomplete data, potentially affecting the identification of anaphylaxis cases. Second, the lack of a temporal relationship in some records precluded definitive attribution of anaphylaxis to mastocytosis. Third, the rarity of mastocytosis and a single Centre recruitment limited the sample size, which may impact statistical power and generalizability of the findings. Lastly, in the absence of genetic testing, it was not possible to search for H*α*T, a genetic trait known to increase the risk of severe anaphylaxis when associated with SM and IgE-mediated allergy (1).

## Conclusions

Pre-SM and recognized SM are associated with a higher risk of anaphylaxis compared with the general population. Idiopathic anaphylaxis can occur across most subtypes, while specific triggers exhibit peculiar characteristics: Hymenoptera venom is mainly implicated in IgE-mediated anaphylaxis in indolent forms, whereas drug-induced anaphylaxis is more frequent in advSM subtypes, predominantly through IgE-independent mechanisms.

Patients sensitized to Hymenoptera venom or lacking skin lesions need a close allergological monitoring. In fact, the presence of cutaneous lesions is a protective factor in non-advanced forms, while sensitization to Hymenoptera venom is a significant risk factor and should be a central focus of allergological management.

The findings support the need for personalized allergological workup, as anaphylaxis risk varies significantly based on disease subtype and cutaneous involvement.

Future studies should aim to investigate the prevalence of HαT in different SM subtypes to refine allergological risk stratification.

## Data Availability

The raw data supporting the conclusions of this article will be made available by the authors, without undue reservation.
